# Role of Zn doping in oxidative stress mediated cytotoxicity of TiO_2_ nanoparticles in human breast cancer MCF-7 cells

**DOI:** 10.1038/srep30196

**Published:** 2016-07-22

**Authors:** Maqusood Ahamed, M. A. Majeed Khan, Mohd Javed Akhtar, Hisham A. Alhadlaq, Aws Alshamsan

**Affiliations:** 1 King Abdullah Institute for Nanotechnology, King Saud University, Riyadh, Saudi Arabia; 2 Department of Physics and Astronomy, College of Science, King Saud University, Riyadh, Saudi Arabia; 3 Nanomedicine Research Unit, Department of Pharmaceutics, College of Pharmacy, King Saud University, Riyadh, Saudi Arabia

## Abstract

We investigated the effect of Zn-doping on structural and optical properties as well as cellular response of TiO_2_ nanoparticles (NPs) in human breast cancer MCF-7 cells. A library of Zn-doped (1–10 at wt%) TiO_2_ NPs was prepared. Characterization data indicated that dopant Zn was incorporated into the lattice of host TiO_2_. The average particle size of TiO_2_ NPs was decreases (38 to 28 nm) while the band gap energy was increases (3.35 eV–3.85 eV) with increasing the amount of Zn-doping. Cellular data demonstrated that Zn-doped TiO_2_ NPs induced cytotoxicity (cell viability reduction, membrane damage and cell cycle arrest) and oxidative stress (reactive oxygen species generation & glutathione depletion) in MCF-7 cells and toxic intensity was increases with increasing the concentration of Zn-doping. Molecular data revealed that Zn-doped TiO_2_ NPs induced the down-regulation of super oxide dismutase gene while the up-regulation of heme oxygenase-1 gene in MCF-7 cells. Cytotoxicity induced by Zn-doped TiO_2_ NPs was efficiently prevented by N-acetyl-cysteine suggesting that oxidative stress might be the primarily cause of toxicity. In conclusion, our data indicated that Zn-doping decreases the particle size and increases the band gap energy as well the oxidative stress-mediated toxicity of TiO_2_ NPs in MCF-7 cells.

Nanotechnology offers a great opportunity to develop nano-scale (1–100 nm) materials with unique characteristics. Nanomaterials with unique features such as quantum effect and optical behavior make them suitable for biomedical applications. Being smaller than cellular organelles, nanoparticles (NPs) are better able to penetrate cells and interact with biomolecules where larger particles have limited accessibility^1^,^2^. Metal oxide NPs represent an important class of materials that are generated in high volume and frequently utilized for their semiconductor properties. TiO_2_ NPs are environment friendly, relatively stable, have excellent biocompatibility with low or no toxicity, and low cast^3^,^4^,^5^. These properties make TiO_2_ NP an excellent candidate for biomedical applications such as drug delivery and cancer therapy^6^,^7^.

TiO_2_ NPs possess unique photocatalytic activity^8^,^9^,^10^,^11^. UV light induced electron-hole (e^−^/h^+^) pair generation in TiO_2_ NPs is a major factor for induction of biological response^12^,^13^. However, the intrinsic toxicity of the high energy UV wavelength needed to drive this e^−^/h^+^ pair generation provides a potential obstacle to studying the biological effects of TiO_2_ NPs photoactivation^14^,^15^,^16^,^17^.

A new area of research has been opened upon doping of NPs. Doping of metal oxide NPs with transition metal ions causes significant changes in the behavior of host NPs^18^,^19^,^20^. Sakthivel *et al*.^21^ demonstrated that optical absorption of ZnO can be enhanced by creating more defects (e.g. metal ions doping) on its surface. Our previous work reported that aluminum (Al) doping tunes band gap energy as well as cytotoxicity of ZnO NPs in human cells^22^. Like ZnO, TiO_2_ NP is a conventional wide band-gap semiconductor with tunable properties. However, studies on effect of metal ions doping on physicochemical properties and biological response of TiO_2_ NPs in human cells is largely lacking. This study was designed to investigate the role of Zn-doping on structural and optical behavior as well as the cytotoxic response of TiO_2_ NPs in human breast cancer MCF-7 cells. We further explore the potential mechanisms of cytotoxicity caused by Zn-doped TiO2 NPs in MCF-7 cells through reactive oxygen species (ROS) generation and oxidative stress. We have selected MCF-7 cell line because the breast cancer is a severe and life threatening cancer and the incidence of such type of cancer is increasing at an alarming rate worldwide^22^. This cell line (MCF-7) has also been widely utilized in toxicological and pharmacological studies^23^,^24^.

## Results and Discussion

### TEM study

**Figure 1:**
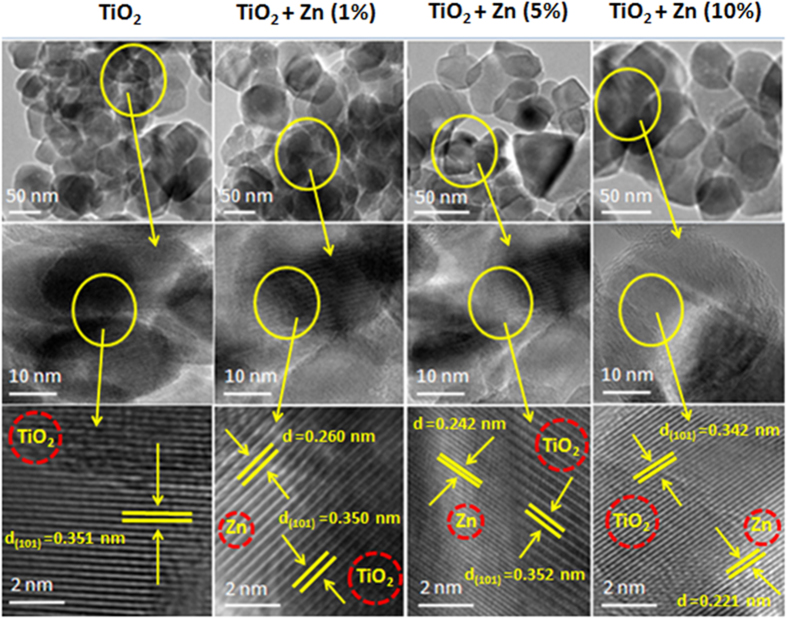
Transmission electron microscopy characterization of pure and Zn-doped TiO_2_ NPs. Upper & middle panels represent the low resolution images while lower panel shows the high resolution images.

**Figure 2:**
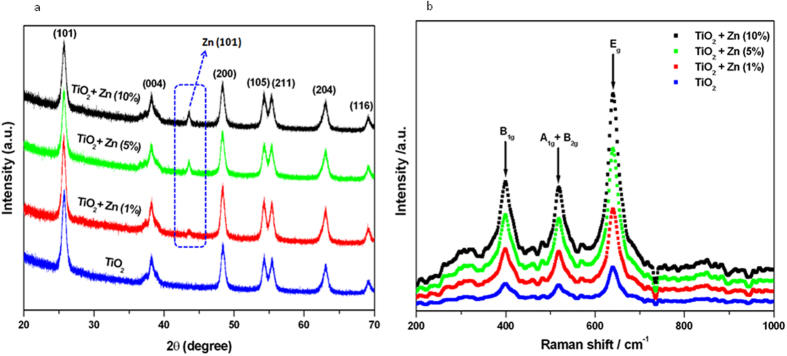
XRD and Raman characterization of pure and Zn-doped TiO_2_ NPs. (**a**) XRD spectra and (**b**) Raman spectra.

Field emission transmission electron microscopy (FETEM) was used to characterize the size of NPs, growth pattern and the distribution of crystallites. Upper and middle panels of Fig. 1 depict the low resolution images of pure and Zn-doped TiO_2_ NPs. These images suggested that particles were agglomerated. The primary particle size was determined from measuring over 100 NPs in random fields of view. The pure and Zn-doped (1–10 at wt%) TiO_2_ NPs with a primary size range from 38 to 28 nm suggest that doping significantly decreases the size distribution of NPs (Table 1). Lower panel of Fig. 1 demonstrates the high resolution TEM images of pure and Zn-doped TiO_2_ NPs. These pictures show the presence of both TiO_2_ and Zn NPs with high quality lattice fringes without any distortion. The estimated interplanar spacing of adjacent lattice fringes of pure and Zn-doped (1, 5 & 10 at wt%) TiO_2_ NPs are 0.351 nm, 0.350 nm, 0.352 nm and 0.342 nm which corresponds to the (101) face of anatase TiO_2_, while a lattice fringes of Zn NPs were 0.260 nm, 0.242 nm and 0.221 nm corresponds to the (101) planes of cubic Zn crystal structure. These lattice distances were in accordance with the X-ray diffraction (XRD) spectra (Fig. 2a). XRD analysis of NPs is given in following section.

Energy dispersive X-ray spectroscopy (EDS) analysis suggested that Ti and O were the main elemental composition in pure TiO_2_ NPs while additional Zn peak was observed in Zn-doped TiO_2_ NPs (Supplementary Fig. S1a,b). These results suggested that prepared samples were devoid of any impurities.

### XRD study

X-ray diffraction (XRD) spectra of pure and Zn-doped TiO_2_ NPs are given in Fig. 2a. The diffraction peaks observed at 25.72°, 38.20°, 48.38°, 54.31°, 55.42°. 63.03° and 69.08° corresponded to the pure anatase phase of TiO_2_ with lattice constant 5 3.7852A° (JCPDS No. 21–1272) and were assigned to the (101), (004), (200), (105), (211), (204) and (116) crystallographic planes. However, Zn-doped TiO_2_ NPs depicts one additional peak at 43.56° (101) associated with the face centered cubic phase of metallic pure Zn (JCPDS PDF #00-004-0831) (Supplementary Fig. S2). Any peaks related to impurities were not detected for pure or Zn-doped TiO_2_ NPs supporting the EDS results. XRD data were also in agreement with high resolution TEM studies (Fig. 1). The broadening diffraction peaks indicate crystals were small in size with semiconducting nature^25^. Moreover, (101) anatase peak exhibited a slight displacement towards a lower 2θ angle for the two higher doping levels (5 & 10% of Zn doping) (Supplementary Fig. S3). Shifting of peak could be due to incorporation of dopant ions (Zn) into the lattice of the host material (TiO_2_ NPs). Similar results were observed in earlier studies^9^,^22^. Using the broadening of (101) anatase peak the TiO_2_ crystallite size was calculated by Scherrer equation^26^. This indicated that size of TiO_2_ NPs decreases with increasing the doping concentrations (Table 1). Reduction in size of metal oxide NPs due doping is a very common trend and also reported in our earlier work^22^. Reduction in the particle size of doped TiO_2_, in comparison to the undoped one, can be attributed to the substitution of Zn ions for TiO_2_ in the crystalline structure. This may be due to the fact that Zn^+2^ ions have an ionic radius of 0.60 Å, which is smaller, compared to ionic radius of 0.68 Å of Ti^4+^ ions. The NPs size calculated from XRD spectra was also in agreement with the TEM results.

### Raman study

To evaluate the phases in pure and Zn-doped TiO_2_ NPs, Raman investigation was performed (Fig. 2b). Raman spectra of pure TiO_2_ NPs exhibit peaks which are located at approximately 398, 520 and 640 cm^−1^. These peaks correspond to the lattice vibratioanal mode model of the B1g, B1g and A1g and Eg modes of anatase TiO_2_ respectively^27^,^28^. Moreover, there is no Zn induced Raman peaks were observed in Zn-doped TiO_2_ samples. The absence of the characteristic Zn vibration modes in the Raman spectra reveals that there is no segregation of Zn particles from TiO_2_ NPs. This indicates that Zn dopants might occupy the substitutional sites in the host TiO_2_ lattice.

### Optical study

**Figure 3:**
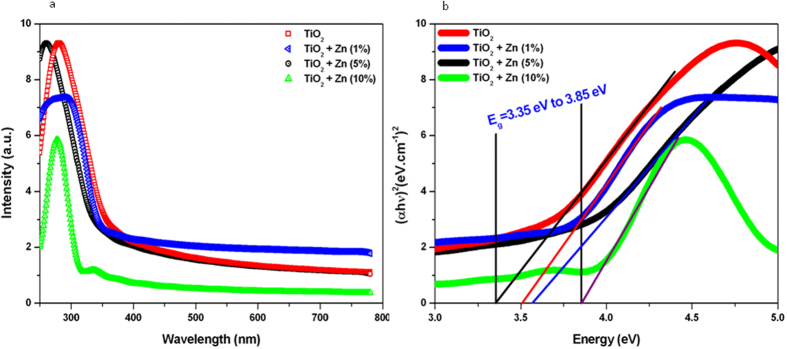
Optical characterization of pure and Zn-doped TiO_2_ NPs. (**a**) UV-visible absorption spectra and (**b**) (αhν)^2^ vs photon energy plots of the corresponding sample used to determine their band gap energy levels.

The optical property of pure and Zn-doped TiO_2_ NPs was characterized by the band gap that is essentially the energy interval between valence band and conduction band, each of which has a high density of states. Band gap energy of metal oxide NPs plays crucial role in their interaction with biological systems^18^,^19^,^20^. The absorption spectra of pure and Zn-doped TiO_2_ NPs are shown in Fig. 3a. Absorption spectra show that there are blue shift of the light absorption edge of Zn-doped TiO_2_ NPs as compared to pure TiO_2_ anatase and the degree of blue shift increases with the increasing amount of Zn dopants. The blue shift of the light absorption is the consequences of the wider band gap energy. TiO_2_ NP is a large band gap semiconductor and the blue shift is attributed to the quantum size effect for semiconductors^29^. This leads to motion of Fermi level towards the conduction band due to an increase in electron concentrations from Zn ions.

In order to evaluate the band gap energy (Eg) of the samples, (αhν)m were plotted against the photon energy (hν), where m is an integer whose value reveals the type of optical transition. In the present study, the nature of the transition was found to be direct, and the energy band gap was calculated by means of following relation^20^.


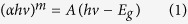


where Eg is the band gap energy, A is constant depending on transition probability and m is the power index that is related to the optical absorption process. Theoretically m equals to 2 or ½ for a direct or an indirect allowed transition, respectively. Here,


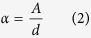


where ‘A’ is the absorbance and d is the thickness of the cuvette. Figure 3b illustrates (αhν)^2^ versus photon energy (hν) plots for pure and Zn-doped TiO_2_ NPs. The band gap energy (Eg) of TiO_2_ NPs increases from 3.31 to 3.87 eV with increasing of the concentration of Zn dopants. This can be explained by the fact that either it may due to any charged defects or the charged defect formed had been neutralized by other defects. Hence, the blue shift in the band gap value by Zn-doping suggest an increase in the n-type carrier concentration, most of the Zn ions must be incorporated as interstitial donors into the structure rather than substitution of acceptors. Our results are in agreement with other studies that reported the tuning of band gap energy level of metal oxides NPs with metal ions doping^19^,^30^,^31^.

### Hydrodynamic size and zeta potential

To get a realistic overview of prepared NPs behavior when they get interaction with the human cells, hydrodynamic size and zeta potential of NPs in deionized water and culture medium was determined. These parameters were determined by Malvern ZetaSizer Nano (Malvern Instruments, UK) as described by Murdock *et al*.^32^ Hydrodynamic size of pure and Zn-doped (1–10%) TiO_2_ NPs was 5–10 times higher as compared to the primary size (size calculated from TEM and XRD) (Table 1). The higher size of NPs in aqueous suspension was due to their tendency of agglomeration. These findings are in agreement with other studies^33^,^34^ and briefly discussed in our previous work^20^. We further observed that hydrodynamic size of NPs was lower in complete cell culture medium (DMEM + 10% FBS) as compared to deionized water (Table 1). Similar drop in the hydrodynamic size of metal and metal oxide NPs in cell culture medium as compared to water was reported in other studies^3^,^35^. This reduction in hydrodynamic size could be attributed to the serum in the culture medium. The serum in the medium is known to rapidly bind to the NPs and form a protein corona around the NPs^5^,^36^. This protein corona further helps the NPs to disperse by providing steric hindrance and electrostatic repulsion between NPs^37^,^38^. The absorption of protein on the NP surface not only affects the size and physical properties of NP but also affects the interaction of NPs with cellular systems.

Zeta potential measurement indicated that all the prepared NPs dispersed in deionized water had positive surface charge. However, the surface charge of the aggregates in complete culture medium was negative (Table 1). This change in zeta potential on the surface of NPs may be explained by the formation of a negatively charged protein corona on the surface of NPs. Therefore, not only the primary size but also the secondary size (hydrodynamic size) and zeta potential of NPs could be used as characteristic parameters in biochemical studies.

In addition, we have utilized pure Zn NPs in cytotoxicity studies. Therefore, characterization data of Zn NPs is given in supplementary Fig. S2.

### Cytotoxic effect of pure and Zn-doped TiO_2_ NPs in MCF-7 cells

**Figure 4:**
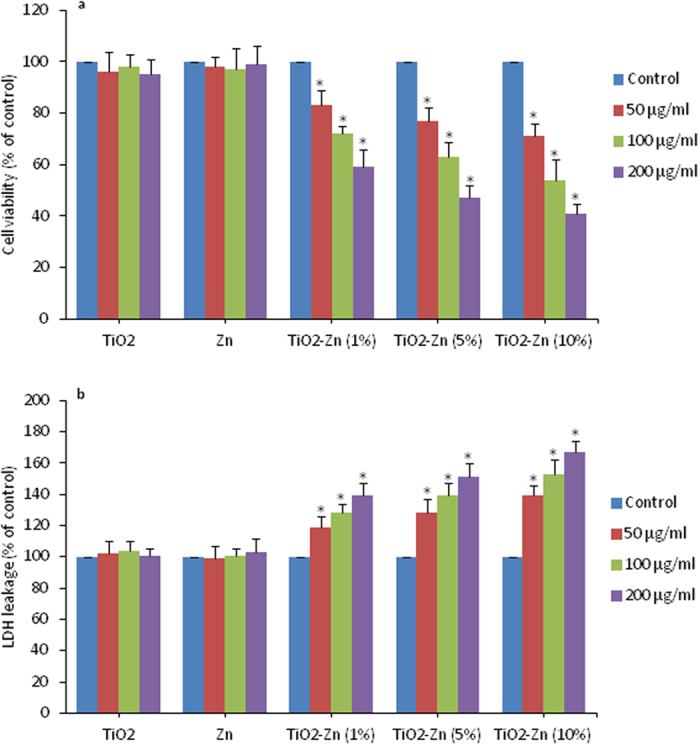
Cytotoxic activity of pure and Zn-doped TiO_2_ NPs in MCF-7 cells. (**a**) Cell viability determined by MTT assay. This assay measures the mitochondrial function by determining the ability of living cells to reduce MTT into blue formazon product. Cells were treated with 50, 100 & 200 μg/ml of pure and Zn-doped TiO_2_ NPs as well as pure Zn NPs for 24 h. Cells not exposed to NPs served as a negative control. (**b**) Lactate dehydrogenase (LDH) assay. LDH is an enzyme widely present in the cytosol that converts lactate into pyruvate. When plasma membrane integrity is disrupted, LDH leaks into culture media and its extracellular level is elevated. Exposure of NPs to cells was similar as in MTT assay. Data represented are mean ± SD of three identical experiments made in three replicate. *Significant difference as compared to control (p < 0.05).

MCF-7 cells were exposed to pure and Zn-doped TiO_2_ NPs as well as pure Zn NPs at the concentrations of 50, 100 and 200 μg/ml for 24 h and cytotoxicity was determined by MTT assay, LDH assay and cell cycle analysis. MTT data demonstrated that Zn-doped TiO_2_ NPs decreased the viability of MCF-7 cells and incremental Zn-doping resulted in lower cell viability (Fig. 4a). Contrary, pure TiO_2_ and Zn NPs did not decrease the viability of MCF-7 cells.

LDH assay demonstrated that Zn-doped TiO_2_ NPs induced membrane damage and LDH leakage effect was incremental with increasing the concentration of Zn-doping (Fig. 4b). However, exposure to pure TiO_2_ and Zn NPs had no effect on LDH leakage in MCF-7 cells. Selection of 50–200 μg/ml dosage range of NPs for cytotoxicity studies was based on a preliminary dose-response study (Supplementary Fig. S4). Prepared library of Zn-doped TiO_2_ NPs did not cause cytotoxicity to MCF-7 cells below the concentration of 50 μg/ml.

We further observed that Zn-doped TiO_2_ NPs induce cell cycle arrest in MCF-7 cells. For example, exposure of Zn-doped (10%) TiO_2_ NPs caused the appearance of 11.9% cells in SubG1 phase as compared to 5.9% cells of control group (Supplementary Fig. S5). On the other hand, pure TiO_2_ and Zn NPs did not cause cell cycle arrest in MCF-7 cells. Overall, our results are in agreement with several earlier reports showing that pure TiO_2_ NPs did not induce cytotoxicity to human cells^3^,^4^,^5^,^11^.

### Oxidant (ROS) and antioxidant (GSH) levels in MCF-7 cells after exposure to pure and Zn-doped TiO_2_ NPs

**Figure 5:**
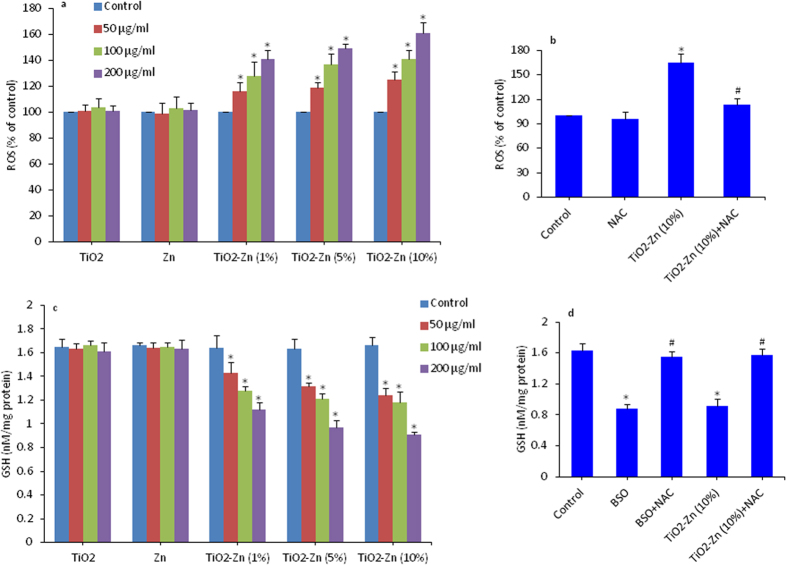
Oxidant ROS and antioxidant GSH levels in MCF-7 cells after exposure to pure and Zn-doped TiO_2_ NPs. (**a**) Intracellular generation of ROS was measured by 2,7-dichlorofluorescin diacetate (DCFH-DA) fluorescence-based assay. Cells were treated with 50, 100 & 200 μg/ml of pure and Zn-doped TiO_2_ NPs as well as pure Zn NPs for 6 h. Cells not exposed to NPs served as a negative control. (**b**) Intracellular ROS levels in the presence or absence of N-acetyl cysteine (NAC). (**c**) GSH level was determined by Ellman’s reagent. Exposure of NPs to cells was similar as in ROS assay. (**d**) Intracellular GSH levels in the presence or absence of buthionine sulphoximine (BSO) and NAC. The BSO is an inhibitor of GSH biosynthesis. GSH levels were expressed in terms of nmole/mg protein. Data represented are mean ± SD of three identical experiments made in three replicate. *Significant difference as compared to the control (p < 0.05). ^#^Significant inhibitory effect of NAC on ROS generation and GSH depletion (p < 0.05).

Oxidative stress plays a critical role in toxicity of nano-scale materials whether by the excessive production of reactive oxygen species (ROS) or by depletion of cellular antioxidant levels^1^. Metal oxide NPs has potential to induce oxidative damage to cellular components^23^,^39^,^49^. We studied the intracellular ROS generation in MCF-7 cells by exposure to 50, 100, and 200 μg/ml of pure and Zn-doped TiO_2_ as well as Zn NPs for 6 h. ROS level was measured by the fluorescence-based assay. Results have shown that increased ROS level was dose-dependent and proportional to the concentration of Zn-doping in TiO_2_ NPs (Fig. 5a). However, pure TiO_2_ and Zn NPs did not induce ROS generation. Besides, treatment with N-acetyl-cysteine (NAC), an ROS scavenger significantly prevented the ROS generation induced by Zn-doped TiO_2_ NPs (Fig. 5b).

Excessive generation of ROS is potentially toxic to cells because of their ability to oxidize a range of biomolecules, including the glutathione (GSH), which plays an important role in maintaining cellular redox homeostasis through its antioxidant effects. Exposure to pure and Zn-doped TiO_2_ NPs at 50, 100, and 200 μg/ml for 6 h induced a dose-dependent depletion of GSH, proportional to the amount of Zn-doping (Fig. 5c). Contrary, exposure of pure TiO_2_ and Zn NPs did not cause GSH depletion. As a positive control buthionine sulphoximine (BSO) also induced GSH depletion in MCF-7 cells. BSO is a well-known inhibitor of c-glutamyl-cysteine synthetase in the pathway of GSH biosynthesis^41^. In addition, treatment with NAC restored cellular GSH in cells challenged with Zn-doped TiO_2_ NPs or BSO. Antioxidant NAC is known to replenish cellular GSH preventing the cell death^42^. Moreover, we also observed that NAC effectively prevented the cytotoxic effects of MCF-7 cells caused by Zn-doped TiO_2_ (Supplementary Fig. S6).

### Effect of pure and Zn-doped TiO_2_ NPs on SOD and HO-1 levels in MCF-7 cells

**Figure 6:**
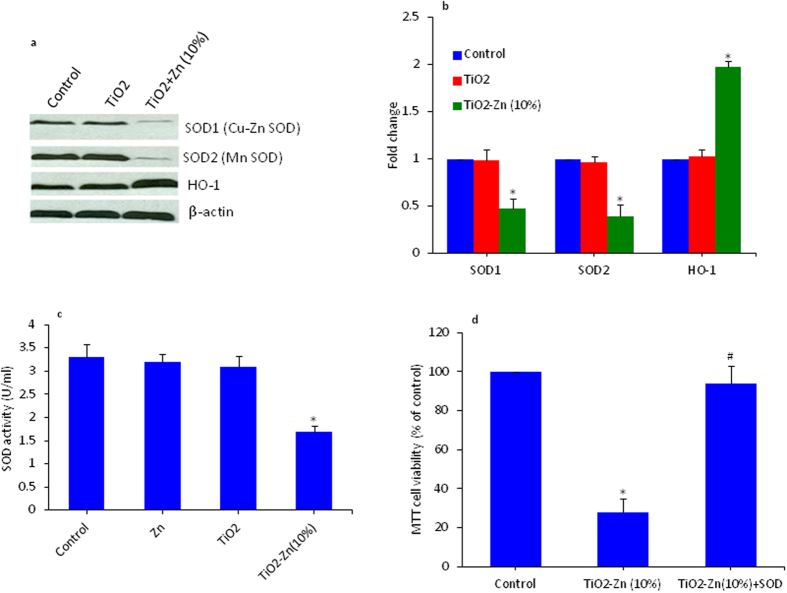
Effect of pure and Zn-doped TiO_2_ NPs on superoxide dismutase (SOD) and heme oxygenase 1 (HO-1) genes in MCF-7 cells. (**a**) Western blot analysis of SOD1 (CuZn-SOD), SOD2 (Mn-SOD) and HO-1 protein levels. Cells were treated with 200 μg/ml pure and Zn-doped TiO_2_ NPs for 6 h. Cells not exposed to NPs served as a negative control. The treated and untreated cells were lysed in RIPA buffer and cell extract subjected to western blots with anti-SOD1, anti-SOD2 & anti-HO-1 antibodies. The β-actin blot is a loading control. (**b**) Protein levels were also analyzed by desitometric analysis using AlphaEase TM FC StandAlone V.4.0.0 software. Results are expressed as a fold change over the control group. (**c**) SOD enzyme activity in MCF-7 cells after exposure to pure and Zn-doped TiO_2_ NPs. Cells were treated with 200 μg/ml of pure and Zn-doped TiO_2_ NPs as well as pure Zn NPs for 6 h. SOD activity was expressed in terms of U/ml. (**d**) SOD enzyme extract attenuates Zn-doped TiO_2_ NPs induced cytotoxicity. Cells were treated with 200 μg/ml of Zn-doped TiO_2_ NPs in the presence or absence of SOD enzyme extract. Data represented are mean ± SD of three identical experiments made in three replicate. *Significant difference as compared to the control (p < 0.05). ^#^Significant inhibitory effect of SOD on cell viability reduction (p < 0.05).

Superoxide dismutase (SOD) enzyme is a first line of the antioxidant defense system. SOD converted superoxide (O_2_^•−^) radicals into hydrogen peroxides (H_2_O_2_). In human cells, there are three forms of SOD: cytosolic Cu/Zn SOD (SOD1), mitochondrial MnSOD (SOD2) and extracellular SOD^43^. We have utilized Western blotting technique to examine the protein levels of SOD1 & SOD2 in MCF-7 cells exposed to pure and Zn-doped (10%) TiO_2_ NPs at a concentration of 200 μg/ml for 6 h. Results showed that Zn-doped TiO_2_ NPs decreased the protein level of SOD 1 and SOD2 (Fig. 6a,b). However, pure TiO_2_ NPs did not produce any effect on the protein levels of either form of SOD.

Western blotting was further used to assess the effect of pure and Zn-doped TiO_2_ NPs on heme oxygenase-1 (HO-1) protein level. We observed increased level of HO-1 protein in MCF-7 cells exposed to 200 μg/ml of Zn-doped (10%) TiO_2_ NPs as compared to control (Fig. 6a,b). However, pure TiO_2_ did not change the expression level of HO-1protein. The HO-1 is a novel enzyme with potent anti-inflammatory, antioxidant, and antiproliferative effects^44^,^45^. The HO-1 is a rate-limiting enzyme that converts heme into biliverdin, releasing free iron and carbon monoxide. Biliverdin is rapidly metabolized to bilirubin, which is a powerful antioxidant. It is likely that HO-1 activity is a component of the cellular defense mechanism against oxidative stress^1^,^19^,^39^.

**Figure 7:**
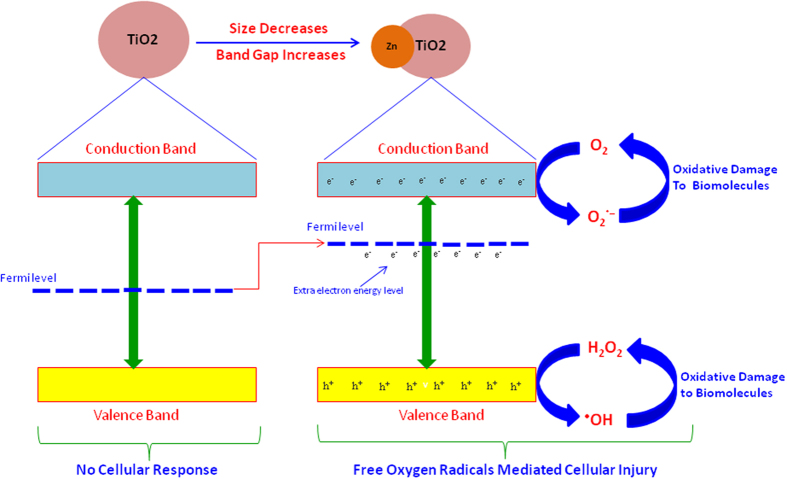
Possible mechanism of cytotoxicity caused by Zn-doped TiO_2_ NPs. Pure TiO_2_ NPs was not able to elicit cellular response because of lack of electrons (e^−^) or holes (h^+^) on the surface of NPs. However, integration of Zn impurities contributes electron energy levels high in semiconductor band gap so that electrons can be easily excited into conduction band, which causes Fermi level to be shifted towards conductions band. Movement of electrons (e^−^) across the band gap to conduction band creates a hole (h^+^) in valence band. The free holes (h^+^) can generate hydroxyl radicals (^•^OH), while the free electrons (e^−^) could lead to formation of superoxide radicals (O_2_^•−^). These free oxygen radicals might be responsible for oxidant injury to cellular systems.

We further investigated the effect of pure and Zn-doped TiO_2_ NPs on the activity SOD enzyme. We found that that Zn-doped (10%) TiO_2_ NPs significantly decreased the activity of SOD enzyme supporting the western blotting results (Fig. 6c). In addition, we studied whether decreased antioxidant level plays a crucial role in the cytotoxicity of Zn-doped TiO_2_ NPs. The MCF-7 cells were exposed to Zn-doped (10%) TiO_2_ NPs in the presence or absence of SOD enzyme extract. Results showed that SOD effectively abrogated the cytotoxicity caused by Zn-doped TiO_2_ NPs (Fig. 7d). These results again suggested that Zn-doped TiO_2_ NPs induced cytotoxicity in MCF-7 cells through the oxidative stress pathway.

### Cellular uptake and dissolution of pure and Zn-doped TiO_2_ NPs in MCF-7 cells

Since metal ion dissolution can lead to metal oxide NPs toxicity, the ionization of pure and Zn-doped TiO_2_ NPs in culture medium was measured as described elsewhere^19^. The NPs in DMEM medium (100 μg/ml) were incubated at 37 °C for 24 h, and the supernatants were collected after centrifugation for acid treatment. Following the quantitative assessment of elemental Ti and Zn content by ICP-MS, it was clear that the Zn NPs had very low rates of dissolution while TiO_2_ NPs did not show any dissolution in culture medium (Supplementary Fig. S7a). Hence, these results rule out the potential cytotoxic activity of NPs through ionic dissolution.

Besides dissolution of metal ions from the surface of NPs, cellular uptake is another factor to consider in cytotoxic activity of metal oxide NPs^18^,^19^. Cellular uptake of pure and Zn-doped TiO_2_ NPs in MCF-7 cells was studied by measuring the cellular Ti content by ICP-MS. After cellular exposure to 100 μg/ml pure and Zn-doped NPs for 24 h, ICP-MS analysis showed the presence of 0.06–0.13 μg Ti/μg protein in MCF-7 cells (Supplementary Fig. S7b).

### Zn-doped TiO_2_ NPs also induced cytotoxicity and oxidative stress in other human cancer cells

In this section, we studied whether Zn-doped TiO_2_ NPs caused cytotoxicity to other human cancer cells. Human lung (A549) and liver (HepG2) cancer cells were treated with pure and Zn-doped (10%) TiO_2_ NPs as well as pure Zn NPs and cytotoxicity and oxidative stress parameters were determined. Results have shown that similar to MCF-7 cells, Zn-doped TiO_2_ NPs induced cell viability reduction, cell membrane damage, ROS generation and GSH depletion in both A549 and HepG2 cells (Supplementary Fig. S8). Pure TiO_2_ and Zn NPs did not induce any toxicity to A549 and HepG2 cells. These observations suggested that Zn-doping provokes cytotoxicity and oxidative response of TiO_2_ NPs in A549 and HepG2 cells in similar fashion as it was in MCF-7 cells.

Taken together, our key finding was that Zn-doping decreases the size while increases the band gap energy level of TiO_2_ NPs. In addition, Zn-doped TiO_2_ NPs induced cytotoxicity and oxidative stress in MCF-7 cells and incremental Zn-doping resulted in more toxicity. The Zn-doping contributes electron energy level high in band gap of TiO_2_, so that electrons can be easily excited into conduction band, which causes Fermi level to be shifted towards conductions band (blue shift). Movement of electrons (e^−^) across the band gap to conduction band creates a hole (h^+^) in valence band. The positive charged (holes h^+^) are powerful oxidants and they can react with H_2_O_2_ or surface-bound chemisorbed hydroxyl group (HO-) to produce hydroxyl (HO^•^) radicals. The conduction band electrons (e^−^) are good reducing agents, and can move to the particle surface and be trapped in metastable surface states, or react with electron acceptors or oxidants such as adsorbed O_2_ to generate superoxide (O_2_^•−^) radicals. These free oxygen radicals react with cellular components and caused oxidant injury (Fig. 7). This might be one of the potential mechanisms of ROS mediated cytotoxicity of Zn-doped TiO_2_ NPs. Increasing of band gap energy with decreasing the size of NPs is also reported by other studies^20^,^22^.

## Conclusion

We investigated the role of Zn-doping in ROS-mediated cytotoxicity of TiO_2_ NPs in MCF-7 cells. We have prepared a library of Zn-doped (1–10 at wt%) TiO_2_ NPs. TEM, XRD and Raman studies suggested that Zn ions were incorporated into the lattice of host TiO_2_. The particle size of TiO_2_ NPs was decreases (38 to 28 nm) while band gap energy was increases (3.35 eV–3.85 eV) with increasing the concentration of Zn-doping. The Zn-doped TiO_2_ NPs were found to induce cytotoxicity and cell cycle arrest in MCF-7 cells and toxic intensity was increases with increasing the level of Zn dopant. The Zn-doped TiO_2_ NPS were also found to induce ROS generation and GSH depletion in a dose-dependent manner and proportional to the level of Zn-doping. Western blotting data revealed that super oxide dismutase (SOD) gene was down-regulated while heme oxygenase-1 gene (HO-1) was up-regulated in MCF-7 cells exposed to Zn-doped TiO_2_ NPs. Furthermore, Zn-doped TiO_2_ NP-cytotoxicity was effectively prevented by N- acetyl-cysteine (NAC) suggesting that ROS generation might be one of the plausible mechanisms of cytotoxicity. Taken together, for the first time we demonstrated that Zn-doping decreases the size and increases the band gap as well the oxidative stress-mediated cytotoxicity of TiO_2_ NPs in MCF-7 cells. This study warrants further investigations to see the effects of metal ions doping on physicochemical behavior as well as the toxic potential of TiO_2_ NPs at *in vivo* level.

## Materials

Dulbecco’s modified eagle’s medium (DMEM), hank’s balanced salt solution (HBSS), fetal bovine serum (FBS) and penicillin-streptomycin were bought from Invitrogen Co. (Carlsbad, CA). N-acetyl cysteine (NAC), 3-(4, 5-dimethylthiazol-2-yl)-2, 5-diphenyltetrazoliumbromide (MTT), 2, 7-dichlorofluorescin diacetate (DCFH-DA), buthionine sulphoximine (BSO), 5,5-dithiobis-(2-nitrobenzoic acid) (DTNB), reduced glutathione (GSH), superoxide dismutase (SOD) and O-phthalaldehyde (OPT) was purchased from Sigma-Aldrich (St. Louis, MO). Anti-SOD1, anti-SOD2, Anti-HO-1 and anti-β-actin antibodies obtained from Cell Signaling Technology Inc (Danvers, MA). Secondary antibodies, RIPA buffer and sodium dodecyl sulphate (SDS) were bought from Santa Cruz Biotechnology Inc (Santa Cruz, CA). All other chemicals utilized in this study were of highest purity available from commercial sources

## Methods

### Synthesis of pure and Zn-doped TiO_2_ NPs

Pure and Zn-doped TiO_2_ NPs were prepared by a simple sol-gel method. Titanium (IV) isopropoxide Ti[OCH(CH_3_)_2_]_4_ and zinc nitrate [Zn(NO_3_)_2_.6H_2_O] were used as precursor materials. In brief, 10 ml of titanium (IV) isopropoxide was dissolved in 50 ml of absolute ethanol. The mixture solution is stirred 15 minutes. The resultant solution is mixed with 10 ml of distilled water and the solution was stirred for 1 h to obtain a clear solution. Now the mixture was transformed in to gel. After aging 24 hours the gel is filtered and dried. Then, prepared TiO_2_ samples were calcined at 450 for 2 h. The Zn-doped TiO_2_ NPs with different amounts of Zinc concentration (1–10 at wt%) were prepared by impregnation of pure TiO_2_ nanopowder suspension in water, with addition of appropriate amount of zinc nitrate while continuous stirring for 1 h. The samples were then filtered, wash and dried at 60 ^o^C for overnight. Then, prepared Zn-doped TiO_2_ samples were calcined at 450 for 2 h.

### Physicochemical characterization of pure and Zn-doped TiO_2_ NPs

The phase purity and crystal structure of pure and Zn-doped TiO_2_ NPs were analysed by XRD instrument (PanAnalytic X’Pert Pro) using Cu-K_α_ radiation (λ = 0.15405 nm, at 45 kV and 40 mA). Transmission electron microscopy (TEM) and high-resolution TEM (HRTEM) were conducted using a JEOL JEM-2100F microscope with an acceleration voltage of 200 kV. The samples were prepared by drop casting a sample dispersed in ethanol on carbon coated Cu-grids and dried in a vacuum. Micro-Raman analysis was carried out at room temperature using a Raman Scope system (JY-Horiba-T64000) in conjunction with a He-Cd Kimmon continuous wave laser operating at a wavelength of 325 nm as the excitation source in the range from 200 cm^−1 ^to 1000 cm^−1^. UV-visible absorption spectra were obtained using a UV-visible spectrophotometer (UV-2550, Shimadzu, Japan) over a wavelength range of 250 nm to 800 nm.

Hydrodynamic size and zeta potential of pure and Zn doped TiO_2_ NPs in deionized water and complete culture medium (DMEM with 10% FBS) was measured by DLS (Nano-ZetaSizer-HT, Malvern, UK) as reported by Murdock and co-workers^32^. In brief, NPs was suspended in deionized water and culture medium at the concentration of 200 μg/ml. This suspension was sonicated at room temperature for 15 minutes at 40 W and studied on DLS. We have chosen 200 μg/ml of NPs concentration for DLS measurement because this was the highest dosage level used in cytotoxicity studies.

### Cell culture

Human breast (MCF-7), lung (A549) and liver (HepG2) cancer cell lines were purchased from American Type Culture Collection (ATCC) (Manassas, VA). Cells were cultured in DMEM medium supplemented with 10% FBS and 100 U/ml penicillin-streptomycin at 5% CO_2_ and 37 °C. At 85% confluence, cells were harvested using 0.25% trypsin and were sub-cultured for biological studies.

### Nanoparticle exposure to cells

Cells were allowed to attach on the surface of culture flask for 24 h before the NPs exposure. Dry powder of NPs was suspended in DMEM medium at a concentration of 1 mg/ml and diluted to appropriate dosages (0.5–200 μg/ml). The different concentrations of NPs were then sonicated at room temperature for 15 min at 40 W to avoid agglomeration of NPs before exposure to cells. In some experiments, cells were pre-exposed for 1 h with NAC (10 μM), BSO (200 μM) or SOD (50 U/ml) before co-exposure with or without NPs.

### Cell viability assay

Cell viability was assessed following the method of Mossman^46^ with few specific changes^40^. This assay measures the mitochondrial function by determining the ability of living cells to reduce MTT into blue formazon product. Briefly, 1 × 10^4 ^cells/well were seeded in 96-well plates and exposed to different concentrations of NPs for 24 h. After the exposure time completed, culture medium was taken out from each well to avoid interference of NPs and replaced with new medium containing MTT solution in an amount equal to 10% of culture volume and incubated for 3 h at 37 °C until a purple-colored formazan product developed. The resulting formazan product was dissolved in acidified isopropanol. Then, 96-well plate was centrifuged at 2300 g for 5 min to settle down the remaining NPs. Further, 100 μl supernatant was transferred to new 96-well plate, and the absorbance was taken at 570 nm utilizing a microplate reader (Synergy-HT, BioTek, Winooski, VT).

### Lactate dehydrogenase assay

LDH assay was carried out using a LDH-cytotoxicity colorimetric assay kit (Bio-Vision Inc., Milpitas, CA). In brief, 1 × 10^4 ^cells/well were seeded in 96-well plates and exposed to different concentrations of NPs for 24 h. After the exposure period had elapsed, each 96-well plate was centrifuged at 2300 g for 5 min to settle the NPs present in the solution. Then 100 μl of the supernatant was transferred to new 96-well plate that already contained 100 μl of the reaction mixture from the BioVision kit and incubated for 30 min at room temperature. At the end of incubation time, absorbance of the solution was measured at 340 nm using the microplate reader (Synergy-HT, BioTek, Winooski, VT). The LDH levels in the culture medium versus those in the cells were quantified and compared with the control values according to the manufacturer of the kit’s instructions.

### ROS assay

Intracellular ROS level was measured using 2,7-dichlorofluorescin diacetate (DCFH-DA) as described by Wang and Joseph^47^ with some specific modifications^40^. Briefly, cells (1 × 10^4 ^cells/well) were seeded in 96-well black-bottomed culture plates and allowed to adhere for 24 h in a CO_2_ incubator at 37 °C. Then, cells were exposed to different concentrations of NPs for various exposure times. At the end of the exposure time, cells were washed twice with HBSS before being incubated in 1 ml of working solution of DCFH-DA at 37 °C for 30 min. Following this, cells were lysed in alkaline solution and centrifuged at 2300 g for 15 min to settle down the cell debris and NPs. A 200 μl supernatant was transferred to a 96-well plate, and fluorescence was measured at 485 nm excitation and 520 nm emission using a microplate reader (Synergy-HT, BioTek, Winooski, VT). The values were expressed as a percent of fluorescence intensity relative to the control cells.

### GSH assay

GSH content was estimated by the fluorometric assay^48^. Cells were treated with different concentrations of NPs for various exposure times. At the end of exposure time, cells were washed with PBS and lysed in distilled water containing 0.1% deoxycholic acid & 0.1% sucrose by four cycles of freeze-thaw and centrifuged at 10,000 g for 10 min at 4 °C. Then, supernatant was precipitated at 0.25% trichloroacetic acid and centrifuged at 10,000 g for 10 min at 4 °C. A 20 μl from the protein precipitated sample was mixed with 160 μl of 0.1 M phosphate-5 mM EDTA buffer, pH 8.3 and 20 μl OPT(1 mg/ml in methanol) in a black 96-well plate. After 2.5 h of incubation at room temperature in the dark, fluorescence was measured at an emission wavelength of 460 nm along with similarly prepared standards of GSH. GSH levels were expressed in terms of nmole/mg protein.

### Western blotting

MCF-7 cells were cultured in 6-well plates and exposed to NPs at the concentration of 200 μg/mL for 6 h. The harvested cell pellets were lysed in RIPA lysis buffer (1X TBS [0.5 MTris-HCl and 1.5 MNaCl] pH 7.4, 1% NP-40, 0.5% sodium deoxycholate, 0.1% SDS, 0.004% sodium azide) in the presence of a protease inhibitor. The cell lysates were then analyzed for protein content using SDS-Page immunoblotting. The membrane was then probed with SOD1, SOD2, HO-1 and β-actin antibodies to determine the level of proteins.

### Superoxide dismutase activity assay

Activity of SOD enzyme was determined using a commercially available kit (Cayman Chemical Company, Ann Arbor, MI). This assay utilizes a tetrazolium salt for detection of superoxide radicals generated by xanthine oxidase and hypoxanthine. Cell extract was prepared for SOD assay. Briefly, cells were cultured in 75-cm^2^ culture flask and exposed to NPs for various exposure times. At the end of the exposure time, cells were harvested in ice-cold phosphate buffer saline by scraping and washed with phosphate buffer saline at 4 °C. The cell pellets were then lysed in cell lysis buffer [1 × 20 mM Tris-HCl (pH 7.5), 150 mM NaCl, 1 mM Na_2_EDTA, 1% Triton, 2.5 mM sodium pyrophosphate]. Centrifugation (15000 g for 10 min at 4 °C) was performed to get supernatant without cell debris and NPs. This supernatant was used for SOD assay as per the manufacturer’s instruction.

### Protein assay

The protein content in cell extract was determined by the Bradford method^49^ using Bradford reagent (Sigma-Aldrich) and bovine serum albumin as the standard.

### Statistical analysis

Statistical analysis was done by one-way analysis of variance followed by Dunnett’s multiple comparison tests. Significance was ascribed at p < 0.05. All analyses were conducted using the Prism software package (GraphPad Software).

## Additional Information

**How to cite this article**: Ahamed, M. *et al*. Role of Zn doping in oxidative stress mediated cytotoxicity of TiO_2_ nanoparticles in human breast cancer MCF-7 cells. *Sci. Rep.* **6**, 30196; doi: 10.1038/srep30196 (2016).

## Supplementary Material

Supplementary Information

## Figures and Tables

**Table 1:** Physicochemical characterization of nanoparticles (mean ± SD, n = 3).

Nanoparticles	TEM size	XRD size	Hydrodynamic sizeα		Zeta potentialα	
			DI Water	CDMEM	DI Water	CDMEM
TiO_2_	38.2 ± 4.3	38.4 ± 2.5	317.2 ± 13.5	233.5 ± 7.8	+19.2 ± 2.1	−15.4 ± 1.3
TiO_2_ + Zn (1%)	35.1 ± 2.6	36.3 ± 3.2	289.3 ± 8.7	227.2 ± 4.2	+18.8 ± 2.7	−16.9 ± 2.2
TiO_2_ + Zn (5%)	31.5 ± 5.7	30.6 ± 1.4	268.9 ± 11.3	218.6 ± 6.5	+18.3 ± 3.2	−16.3 ± 1.5
TiO_2_ + Zn (10%)	28.8 ± 4.5	29.2 ± 2.7	271.7 ± 5.9	203.9 ± 9.4	+17.5 ± 1.8	−17.8 ± 1.7

^α^Particle size and zeta potential in solution were measured by ZetaSizer Nano (Malvern). CDMEM: complete Dulbecco’s modified eagle media, which contains 10% fetal bovine serum (FBS). DI: deionized water.

## References

[b1] Nel A., Xia T., Madler L. & Li N. Toxic potential of materials at the nanolevel. Science 311, 622–627 (2006). 16456071 10.1126/science.1114397

[b2] Jiang W., Kim B. S., Rutka J. T. & Chan W. W. Nanoparticle-mediated cellular response is size-dependent. Nat. Nanotechnol. 3, 145–150 (2008). 18654486 10.1038/nnano.2008.30

[b3] Xia T. . Comparison of the abilities of ambient and manufactured nanoparticles to induce cellular toxicity according to an oxidative stress paradigm. Nano Lett. 6, 1794–1807 (2006). 16895376 10.1021/nl061025k

[b4] Pujalte I. . Cytotoxicity and oxidative stress induced by different metallic nanoparticles on human kidney cells. Part. Fibre Toxicol. 8, 10 (2011). 21371295 10.1186/1743-8977-8-10 PMC3058043

[b5] Setyawati M. I., Tay C. Y. & Leong D. T. Mechanistic investigation of the biological effects of SiO2, TiO2, and ZnO nanoparticles on intestinal cells. Small 11, 3458–3468 (2015). 25902938 10.1002/smll.201403232

[b6] Setyawati M. I. . Titanium dioxide nanomaterials cause endothelial cell leakiness by disrupting the homophilic interaction of VE-cadherin. Nat. Commun. 4, 1673 (2013). 23575677 10.1038/ncomms2655

[b7] Zhao C. . Bio-imaging and photodynamic therapy with tetra sulphonatophenyl porphyrin (TSPP)-TiO2 nanowhiskers: New approaches in rheumatoid arthritis theranostics. Sci. Rep. 5, 11518 (2015). 26153895 10.1038/srep11518 PMC4648397

[b8] Lu N. . Nano-titanium dioxide photocatalytic protein tyrosine nitration: a potential hazard of TiO_2_ on skin. Biochem. Biophys. Res. Commun. 370, 675–680 (2008). 18407831 10.1016/j.bbrc.2008.04.010

[b9] Bhatkhande D. S., Pangarkar V. G. & Beenackers A. M. Photocatalytic degradation for environmental applications-a review. J. Chem. Technol. Biotechnol. 102, 102–116 (2001).

[b10] Choi W., Termin A. & Hoffmann M. R. The role of metal-ion dopants in quantum-sized TiO2: Correlation between photoreactivity and charge-carrier recombination dynamics. J. Phys. Chem. 98, 13669–13679 (1994).

[b11] Brandl F., Bertrand N., Lima E. M. & Langer R. Nanoparticles with photoinduced precipitation for the extraction of pollutants from water and soil. Nat. Commun. 6, 7765 (2015). 26196119 10.1038/ncomms8765 PMC4518270

[b12] Song M. . The *in vitro* inhibition of multidrug resistance by combined nanoparticulate titanium dioxide and UV irradiation. Biomaterials 27, 4230–4238 (2006). 16600364 10.1016/j.biomaterials.2006.03.021

[b13] Li L. . Sub-10 nm rutile titanium dioxide nanoparticles for efficient visible-light-driven photocatalytic hydrogen production. Nat. Commun. 6, 5881 (2015). 25562287 10.1038/ncomms6881

[b14] Hoffmann M. R., Martin S. T., Choi W. & Bahnemann D. W. Environmental applications of semiconductor photocatalysis. Chem Rev. 95, 69–96 (1995).

[b15] Long T. C., Saleh N., Tilton R. D., Lowry G. V. & Veronesi B. Titanium dioxide (P25) produces reactive oxygen species in immortalized brain microglia (BV2): implications for nanoparticle neurotoxicity. Environ. Sci. & Technol. 40, 4346–4352 (2006). 16903269 10.1021/es060589n

[b16] Sinha R. P. & Hader D. P. UV-induced DNA damage and repair: a review. Photochem. & Photobiol. Sci. 4, 225–236 (2002). 12661961 10.1039/b201230h

[b17] George S. . Role of Fe doping in tuning the band gap of TiO_2_ for the photo-oxidation-induced cytotoxicity paradigm. J. Am. Chem. Soc. 133, 11270–11278 (2011). 21678906 10.1021/ja202836s PMC3971840

[b18] Zhang H. . Use of metal oxide nanoparticle band gap to develop a predictive paradigm for oxidative stress and acute pulmonary inflammation. ACS Nano 6, 4349–4368 (2012). 22502734 10.1021/nn3010087 PMC4139054

[b19] Zhang H. . PdO doping tunes band-gap energy levels as well as oxidative stress responses to a Co3O4 p-type semiconductor in cells and the lung. J. Am. Chem. Soc. 136, 6406–6420 (2014). 24673286 10.1021/ja501699e PMC4410908

[b20] Akhtar M. J., Alhadlaq H. A., Alshamsan A., Khan M. M. & Ahamed M. Aluminum doping tunes band gap energy level as well as oxidative stress-mediated cytotoxicity of ZnO nanoparticles in MCF-7 cells. Sci. Rep. 5, 13876 (2015). 26347142 10.1038/srep13876 PMC4561961

[b21] Sakthivela S. . Solar photocatalytic degradation of azo dye: comparison of photocatalytic efficiency of ZnO and TiO_2_. Sol. Energ. Mat. Sol. Cells 77, 65–82 (2003).

[b22] Khan M. M., Kumar S., Khan M. N., Ahamed M. & Al-Dwayyan A. S. Microstructure and blue-shift in optical band gap of nanocrystalline AlxZn1-xO thin films. J. Lumin. 155, 275–281 (2014).

[b23] Akhtar M. J. . Zinc oxide nanoparticles selectively induce apoptosis in human cancer cells through reactive oxygen species. Int. J. Nanomedicine 7, 845–857 (2012). 22393286 10.2147/IJN.S29129 PMC3289443

[b24] Siddiqui M. A. . Copper oxide nanoparticles induced mitochondria mediated apoptosis in human hepatocarcinoma cells. PLoS One 8, e69534 (2013). 23940521 10.1371/journal.pone.0069534 PMC3734287

[b25] Yeha C. L., Yeh S. H. & Ma H. K. Flame synthesis of titania particles from titanium tetraisopropoxide in premixed flames. Powder Technol. 145, 1–9 (2004).

[b26] Choi Y. J., Seeley Z., Bandyopadhyay A., Bose S. & Akbar S. A. Aluminum-doped TiO_2_ nano-powders for gas sensors. Actuat. B Chem. 124, 111–117 (2007).

[b27] Giarola M. . Vibrational dynamics of anatase TiO_2_: Polarized Raman spectroscopy and ab initio calculations. Phys. Rev. B 81, 174305 (2010).

[b28] Tian F., Zhang Y. P., Zhang J. & Pan C. X. Raman spectroscopy: a new approach to measure the percentage of anatase TiO_2_ exposed (001) facets. J. Phys. Chem. C 116, 7515–7519 (2012).

[b29] Henlein A. Small-particle research: physicochemical properties of extremely small colloidal metal and semiconductor particles. Chem. Rev. 89, 1861 (1989).

[b30] Thurber A. . Improving the selective cancer killing ability of ZnO nanoparticles using Fe doping. Nanotoxicology 6, 440–452 (2011). 21635174 10.3109/17435390.2011.587031

[b31] Xiong H. M., Xu Y., Ren Q. G. & Xia Y. Y. Stable aqueous ZnO@polymer core-shell nanoparticles with tunable photoluminescence and their application in cell imaging. J. Am. Chem. Soc. 130, 7522–7523 (2008). 18498161 10.1021/ja800999u

[b32] Murdock R. C., Braydich-Stolle L., Schrand A. M., Schlager J. J. & Hussain S. M. Characterization of nanomaterial dispersion in solution prior to *in vitro* exposure using dynamic light scattering technique. Toxicol. Sci. 101, 239–253 (2008). 17872897 10.1093/toxsci/kfm240

[b33] Bai W. . Toxicity of zinc oxide nanoparticles to zebrafish embryo: a physicochemical study of toxicity mechanism. J. Nanopart. Res. 12, 1645–1654 (2009).

[b34] Sharma V. . DNA damaging potential of zinc oxide nanoparticles in human epidermal cells. Toxicol. Lett. 185, 211–218 (2009). 19382294 10.1016/j.toxlet.2009.01.008

[b35] Ng K. W. . The role of the tumor suppressor p53 pathway in the cellular DNA damage response to zinc oxide nanoparticles. Biomaterials 32, 8218–8225 (2011). 21807406 10.1016/j.biomaterials.2011.07.036

[b36] Lundqvist M. . Nanoparticle size and surface properties determine the protein corona with possible implications for biological impacts. Proc. Natl. Acad. Sci. USA 105, 14265–14270 (2008). 18809927 10.1073/pnas.0805135105 PMC2567179

[b37] Ji Z. . Dispersion and stability optimization of TiO_2_ nanoparticles in cell culture media. Environ. Sci. Technol. 44, 7309–7314 (2010). 20536146 10.1021/es100417s PMC3971839

[b38] Chia S. L., Tay C. Y., Setyawati M. I. & Leong D. T. Decoupling the direct and indirect biological effects of ZnO nanoparticles using a communicative dual cell-type tissue construct. Small 12, 647–57 (2016). 26670581 10.1002/smll.201502306

[b39] Xia T. . Comparison of the mechanism of toxicity of zinc oxide and cerium oxide nanoparticles based on dissolution and oxidative stress properties. ACS Nano 2, 2121–2134 (2008). 19206459 10.1021/nn800511k PMC3959800

[b40] Ahamed M. . Oxidative stress mediated apoptosis induced by nickel ferrite nanoparticles in cultured A549 cells. Toxicology 28, 101–108 (2011). 21382431 10.1016/j.tox.2011.02.010

[b41] Patzewitz E. M., Wong E. H. & Muller S. Dissecting the role of glutathione biosynthesis in Plasmodium falciparum. Mol. Microbiol. 83, 304–318 (2012). 22151036 10.1111/j.1365-2958.2011.07933.x PMC3321222

[b42] Franco R., Panayiotidis M. I. & Cidlowski J. A. Glutathione depletion is necessary for apoptosis in lymphoid cells independent of reactive oxygen species formation. J. Biol. Chem. 282, 30452–30465 (2007). 17724027 10.1074/jbc.M703091200 PMC2267748

[b43] Sandstrom J., Nilsson P., Karlsson K. & Marklund S. L. 10-Fold increase in human plasma extracellular superoxide dismutase content caused by a mutation in heparin-binding domain. J. Biol Chem. 269, 19163–19166 (1994). 8034674

[b44] Araujo J. A., Zhang M. & Yin F. Heme oxygenase-1, oxidation, inflammation, and atherosclerosis. Front. Pharmacol. 3, 119 (2012). 22833723 10.3389/fphar.2012.00119 PMC3400084

[b45] Galeotti C. . Heme oxygenase-1 is dispensable for the anti-inflammatory activity of intravenous immunoglobulin. Sci. Rep. 6, 19592 (2016). 26796539 10.1038/srep19592 PMC4726216

[b46] Mossman T. Rapid colorimetric assay for cellular growth and survival: Application to proliferation and cytotoxicity assays. J. Immunol. Methods 65, 55–63 (1983). 6606682 10.1016/0022-1759(83)90303-4

[b47] Wang H. & Joseph J. A. Quantifying cellular oxidative stress by dichlorofluorescin assay using microplate reader. Free Radic. Biol. Med. 27, 612–616 (1999). 10490282 10.1016/s0891-5849(99)00107-0

[b48] Hissin P. J. & Hilf R. A fluorometric method for determination of oxidized and reduced glutathione in tissues. Anal. Biochem. 74, 214–226 (1976). 962076 10.1016/0003-2697(76)90326-2

[b49] Bradford M. M. A rapid and sensitive method for the quantitation of microgram quantities of protein utilizing the principle of protein-dye binding. Anal. Biochem. 72, 248–254 (1976). 942051 10.1016/0003-2697(76)90527-3

