# Supplementary material for: Role of Zn doping in oxidative stress mediated cytotoxicity of TiO2 nanoparticles in human breast cancer MCF-7 cells
Source: Sci Rep. 2016 Jul 22;6:30196. doi: 10.1038/srep30196 (PMC4957127; doi:10.1038/srep30196)
Supplement: Supplementary Information [file srep30196-s1.pdf]

Supplementary Information

Role of Zn doping in oxidative stress mediated cytotoxicity of TiO_2 nanoparticles in human breast cancer MCF-7 cells

Maqusood Ahamed [a,*], M.A. Majeed Khan [a], Mohd Javed Akhtar [a], Hisham A. Alhadlaq [a,b], Aws Alshamsan [a,c]

[a] King Abdullah Institute for Nanotechnology, King Saud University, Riyadh, Saudi Arabia

[b] Department of Physics and Astronomy, College of Science, King Saud University, Riyadh, Saudi Arabia

[c] Nanomedicine Research Unit, Department of Pharmaceutics, College of Pharmacy, King Saud University, Riyadh, Saudi Arabia

[*]Corresponding author:

Dr. Maqusood Ahamed

Assistant Professor

King Abdullah Institute for Nanotechnology

King Saud University

Riyadh-11451, Saudi Arabia

Email: maqusood@gmal.com (MA)

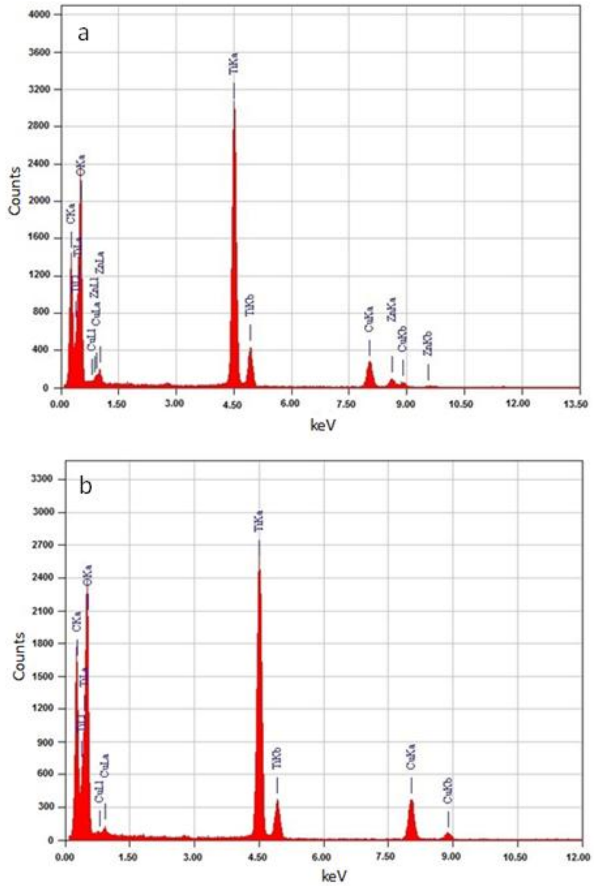

Supplementary Figure S1: Energy dispersive X-ray spectroscopy (EDS) analysis of pure and Zn-doped TiO_2 NPs. It is clear from these images that Ti and O were the main elemental species in pure TiO_2 NPs while additional Zn peaks were observed in Zn-doped TiO_2 NPs. No other element impurity was detected. The peaks of Cu and C observed in the spectra are attributed to the carbon-coated copper TEM grid. EDS spectrum of pure TiO_2 NPs (a) Zn-doped TiO_2 NPs (b)

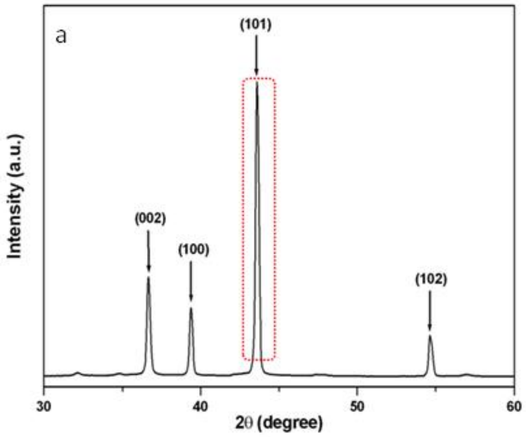

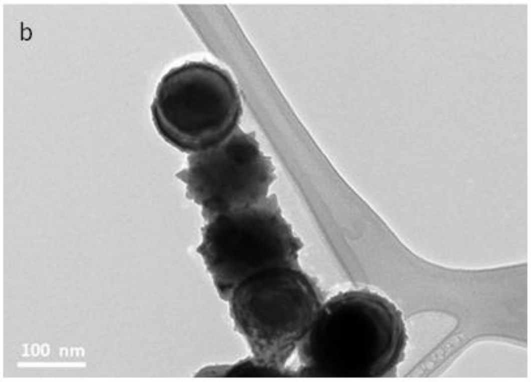

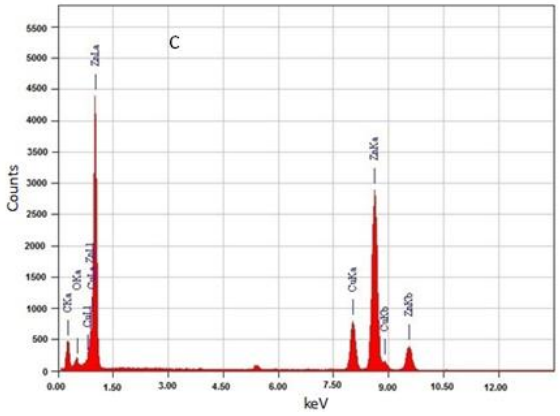

d

Physicochemical characterization of Zn NPs (Mean±SD, n=3)

XRD size	63.3±5.1 nm
TEM size	62.6±4.3 nm
Elemental impurities (EDS)	Not detected
Hydrodynamic size in water	335.5±21.7 nm
Hydrodynamic size in CDMEM	268.4±33.5 nm
Zeta potential in Water	+21.4±2.48 mV
Zeta potential in CDMEM	-17.3±3.33 mV

Supplementary Figure S2: Characterization of Zn NPs. (A) XRD spectra, (B) TEM image, (C) EDS spectra and (D) Table represents summary of characterization data including hydrodynamic size and zeta potential of Zn NPs

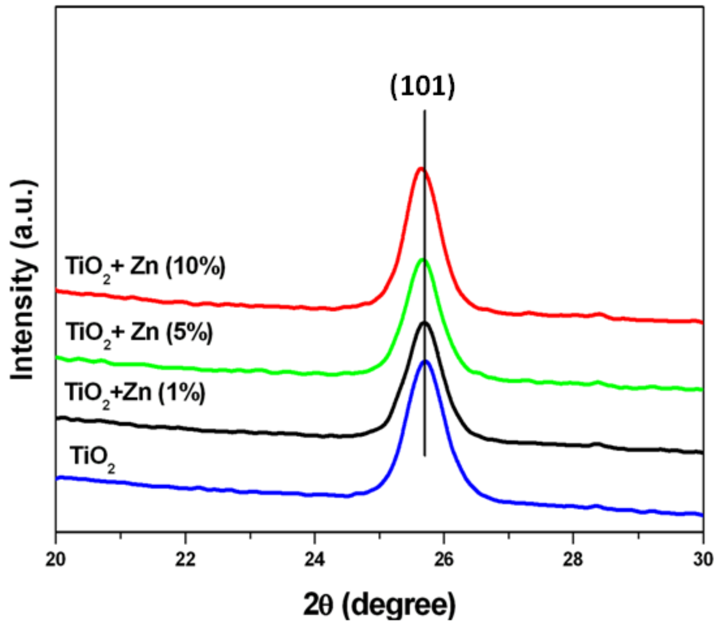

Supplementary Figure S3: XRD pattern (zoom) corresponding to characteristic peak (101) of pure and Zn-doped TiO_2 NPs. The characteristic peak (101) of TiO_2 shifted slightly to lower angle at higher level of Zn-doping (5 & 10%). Shifting of peak could be due to incorporation of dopant ions into the lattice of the host material.

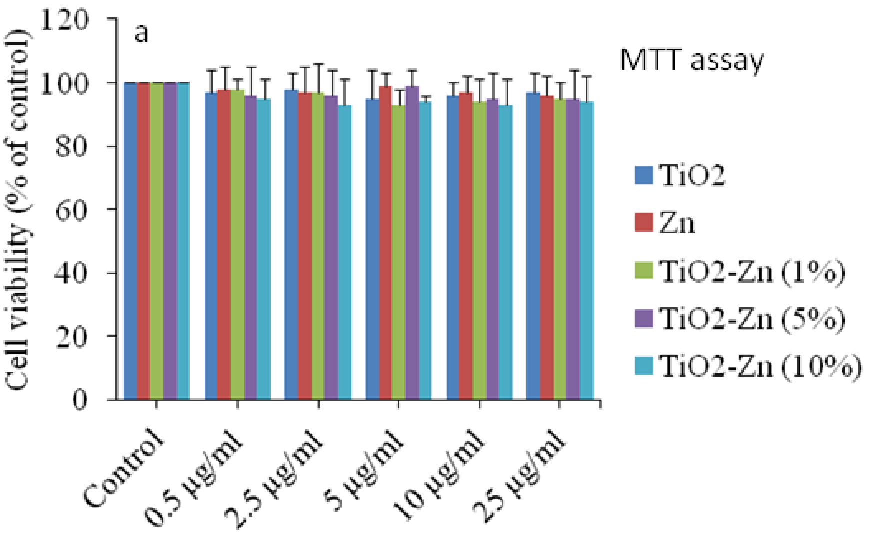

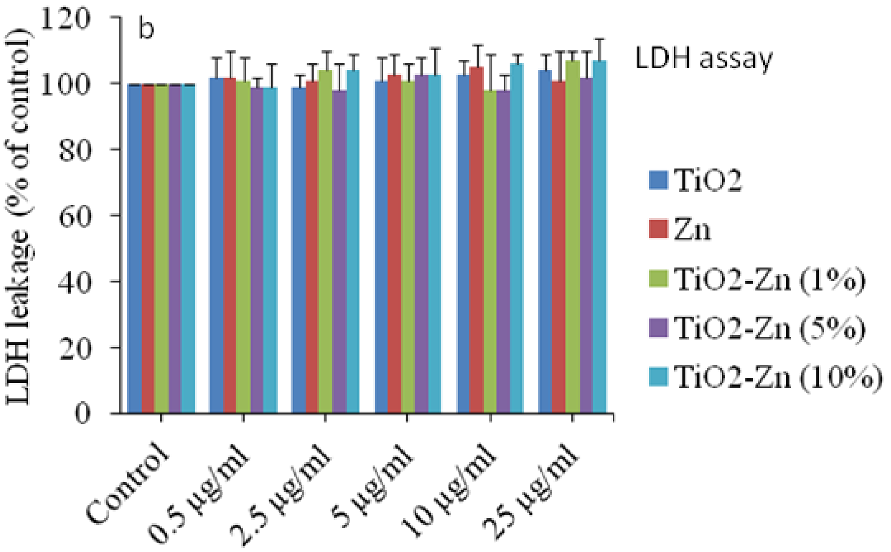

Supplementary Figure S4: Cytotoxicity in MCF-7 cells. (a) Cell viability by MTT assay. Cells were treated with 0.5, 2.5, 5, 10, 20 & 40 µg/ml of pure and Zn-doped TiO_2 NPs as well as pure Zn NPs for 24 h. Untreated cells were used as a negative control. (b) Cell membrane damage by LDH assay. Cellular exposure to the NPs was similar as in a. All the prepared NPs did not induced cytotoxicity to MCF-7 cells up to the concentration of 40 µg/ml. Data represented are mean±SD of three identical experiments made in three replicate. *Significant difference as compared to the control ($p<0.05$).

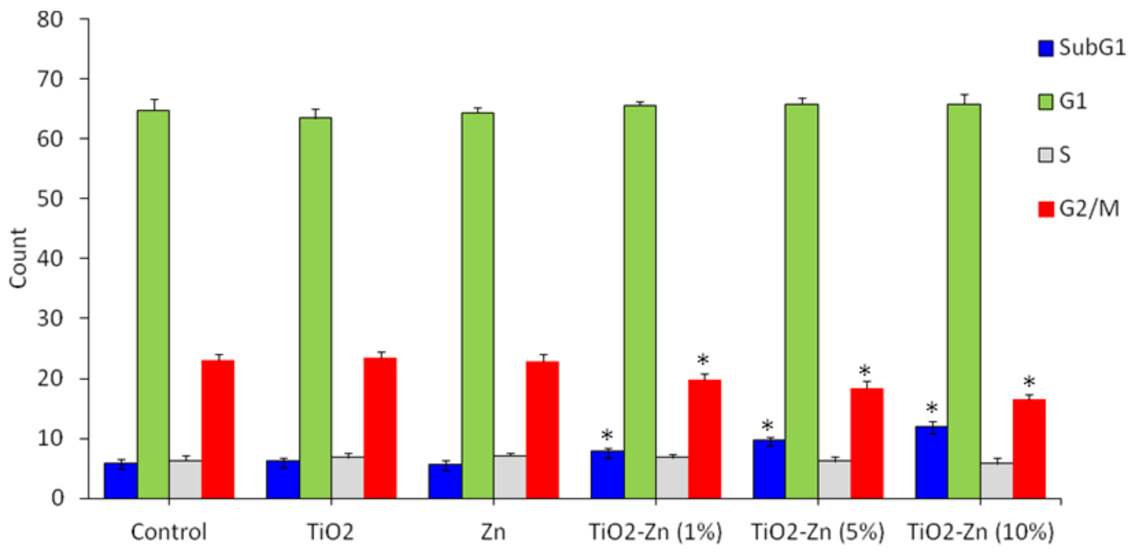

Supplementary Figure S5: Cell cycle analysis in MCF-7 cells. Cells were treated with 200 µg/ml of pure and Zn-doped TiO_2 NPs as well as pure Zn NPs for 24 h. Untreated cells were used as a negative control. Cells with damaged DNA will accumulate in gap1 (G1), DNA synthesis (S), or in gap2/mitosis (G2/M) phase. Cells with irreversible damage will undergo apoptosis, giving rise to accumulation of cells in subG1 phase. We observed that exposure of Zn-doped TiO_2 resulted in the appearance of significant 11.9% cells in SubG1 phase in exposed cells in comparison of 5.9% cells of control group. Statistically significant decline in G2/M phase was also noticeable from the appearance of 19.8, 18.3 & 16.5% of cells in G2/M phase treated with 200 µg/ml of TiO_2-Zn(1%), TiO_2-Zn(5%) & TiO_2-Zn(10%) NPs, respectively versus 23% of cells in G2/M phase of control group. In contrast, pure TiO_2 and Zn NPs did not induce cell cycle arrest in MCF-7 cells. Data represents mean±SD values of different phases of cell cycle obtained from three identical experiments made in three replicate. *Significant difference as compared to control ($p<0.05$).

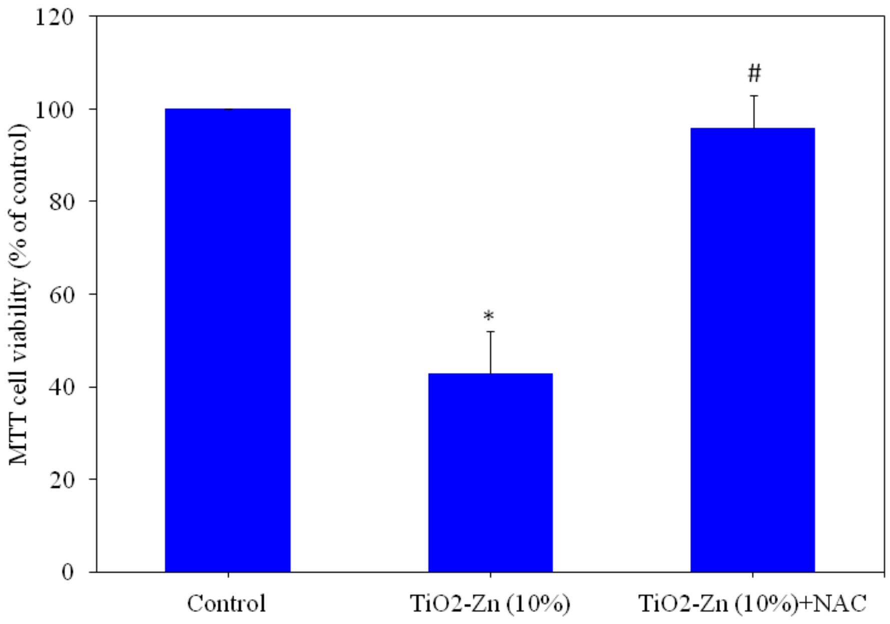

Supplementary Figure S6: N-acetyl cysteine (NAC) effectively prevented the cytotoxicity of MCF-7 cells caused by Zn-doped TiO_2 NPs. NAC is a ROS scavenger. Cells were exposed to Zn-doped TiO_2 NPs at the concentration of 200 μg/ml for 24 h in presence or absence of NAC. Data represented are mean±SD of three identical experiments made in three replicate. *Significant difference as compared to the control ($p<0.05$). [#] Significant inhibitory effect of NAC on MTT cell viability reduction ($p< 0.05$).

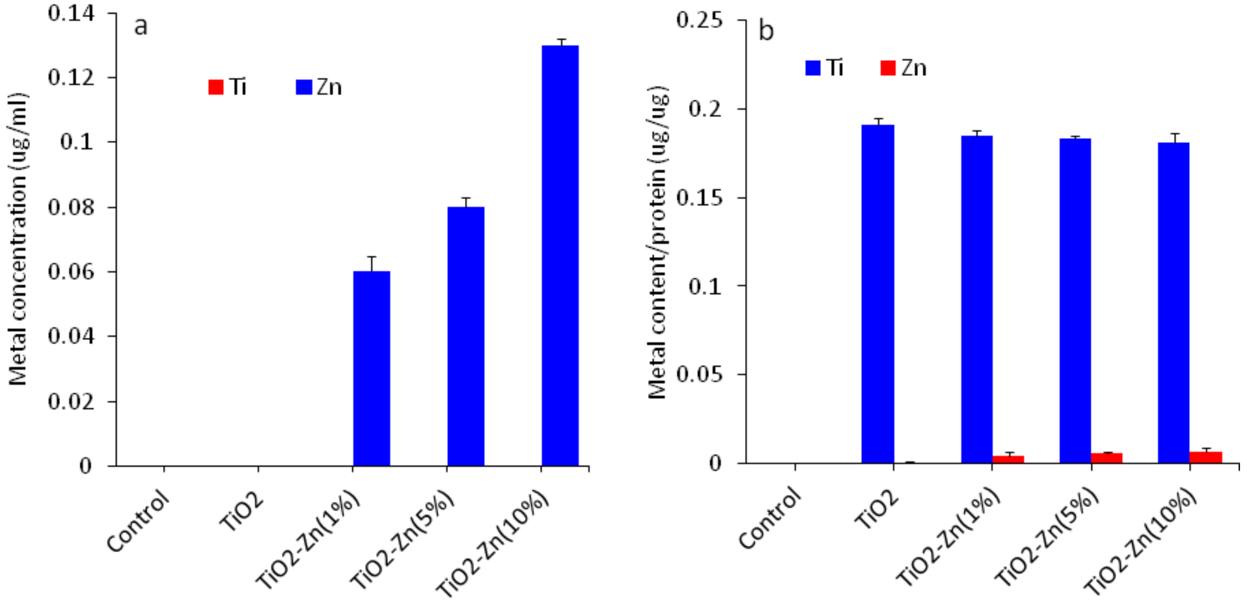

Supplementary Figure S7: Metal dissolution and cellular uptake in MCF-7 cells. (a) Metal dissolution in DMEM culture medium. A 200 µg/ml of pure or Zn-doped TiO2 NPs were incubated at 37 °C for 24 h. Supernatants were collected and elemental Ti or Zn concentrations determined by ICP-MS. (b) ICP-MS was used for measurement of cellular Ti and Zn content in MCF-7 cells. Cells were treated with 50 µg/ml of NPs for 24 h, and untreated cells were used as a control. After PBS washing, cells were harvested using 0.05% trypsin and the cell suspension digested with concentrated nitric acid at 90 °C for 4 h. The well-digested solution was used for ICP-MS measurement.

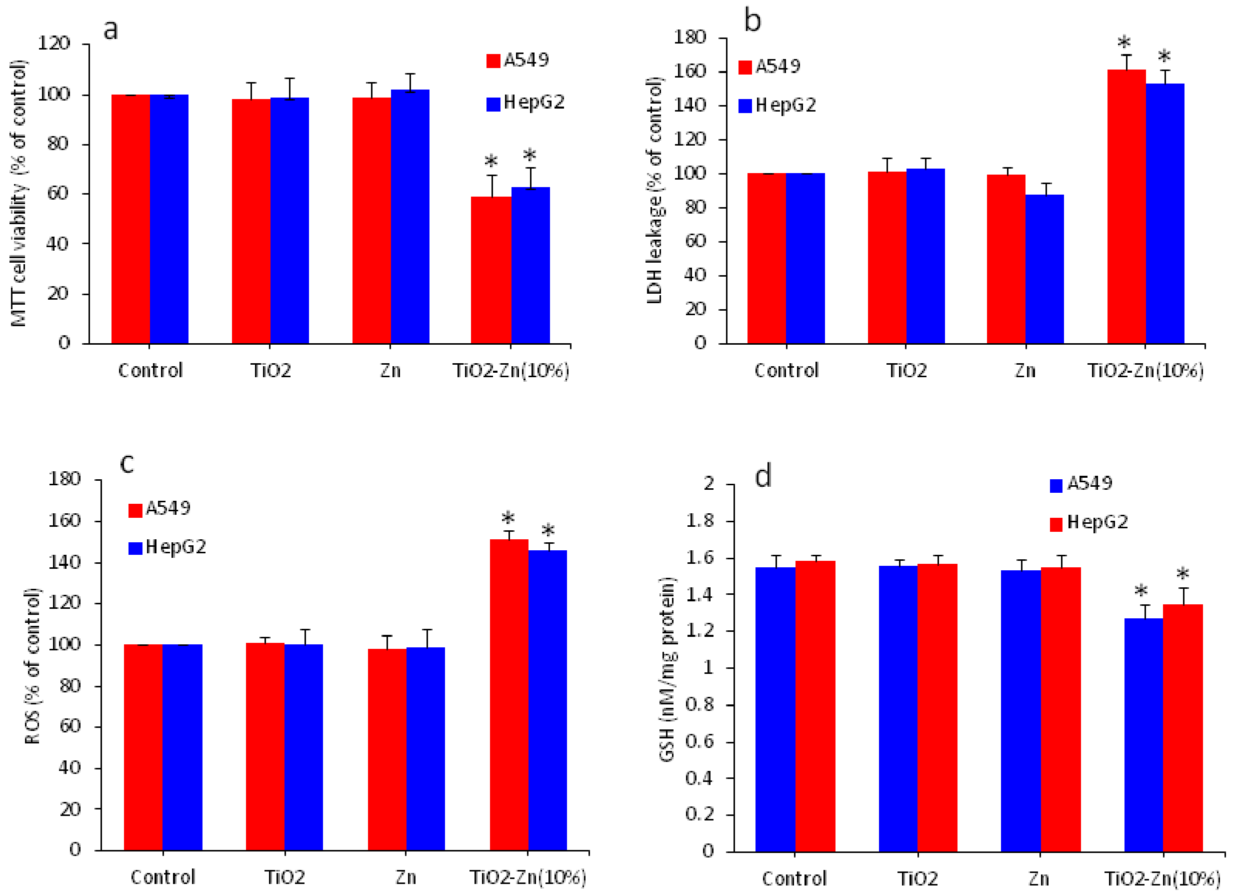

Supplementary Figure S8: Zn-doped TiO_2 NPs induced cytotoxicity and oxidative stress in human lung (A549) and liver (HepG2) cancer cells. Cells were treated with 200 µg/ml of pure and Zn-doped TiO_2 NPs as well as pure Zn NPs for 24 h. Untreated cells were used as a negative control. (a) Cell viability by MTT assay. (b) Cell membrane damage by LDH assay. (c) Intracellular ROS level. (d) Intracellular GSH level. Data represented are mean±SD of three identical experiments made in three replicate. *Significant difference as compared to the control ($p<0.05$).

Methods

Cell cycle analysis. Cells were treated with 200 μg/ml of pure and Zn-doped TiO_2 NPs as well as pure Zn NPs for 24 h. Untreated cells were used as a negative control. At the end of exposure time, cells were harvested and centrifuged at 1000g for 4 min. Pellets were re-suspended in 500 µl of PBS. Cells were fixed with equal volume of chilled 70 % ice-cold ethanol, and incubated at 4 ^{0}C for 1 h. After two successive washes with PBS at 2000g for 4 min, cell pellets were re-suspended in PBS and stained with 50 µg propiodium iodide (PI)/ml containing 0.1% Triton X-100 and 0.5 mg/ml RNAase A for 1 h at 30 °C in dark. Fluorescence of the PI was measured by a Beckman Coulter Flow cytometer (Coulter Epics XL/Xl-MCL, Miami, USA) through a FL-4 filter (585 nm) and 10,000 events were acquired [24]. The data were analyzed by Coulter Epics XL/XL-MCL, System II Software, version 3. Cell debris was characterized by a low FSC/SSC was excluded from the analysis.

Metal dissolution in cell culture medium and cellular uptake of NPs. Ti and Zn dissolution for 0%, 1%, 5% & 10% Zn-doped TiO2 NPs incubated in cell culture medium was determined by ICP-MS. A suspension 100 µg/ml of pure and Zn-doped TiO2 NPs was prepared in DMEM medium and incubated for 24 h at 37 °C with gentle shaking. Then, aliquots from the supernatant were collected and solid components were removed by centrifugation. Then, supernatant was digested by nitric acid at 90 °C for 3 h. The digested solution was dried by evaporation at 120 °C, and 5% nitric acid was added for inductively coupled plasma mass spectrometry (ICP-MS) measurement. The mean±SD of triplicate measures was reported for all dissolution measurements.

Cellular uptake of 0%, 1%, 5% & 10% Zn-doped TiO2 NPs in MCF-7 was measured by ICP-MS. In brief, 5×10^4 cells/well were seeded in 6-well plate and allowed to attach on the surface for 24 h in a CO_2 incubator at 37°C. Cells were treated with 100 μg/ml NPs for 24 h. At the end of exposure time, cells were gently washed three times with PBS and harvested. Cell suspension was digested by concentrated nitric acid at 90 °C for 3 h. The well-digested solution was dried by evaporation at 120 °C, and 5% nitric acid was added for ICP-MS measurement.
